# Hydrogen Sulfide-Mediated Activation of *O*-Acetylserine (Thiol) Lyase and l/d-Cysteine Desulfhydrase Enhance Dehydration Tolerance in *Eruca sativa* Mill

**DOI:** 10.3390/ijms19123981

**Published:** 2018-12-11

**Authors:** M. Nasir Khan, Fahad M. AlZuaibr, Asma A. Al-Huqail, Manzer H. Siddiqui, Hayssam M. Ali, Mohammed A. Al-Muwayhi, Hafiz N. Al-Haque

**Affiliations:** 1 Department of Biology, Faculty of Science, University of Tabuk, Tabuk 71491, Saudi Arabia; falzuaiber@ut.edu.sa; 2 Chair of Climate Change, Environmental Development and Vegetation Cover, Department of Botany and Microbiology, College of Science, King Saud University, Riyadh 11451, Saudi Arabia; aalhuquail@ksu.edu.sa (A.A.A.); manzerhs@yahoo.co.in (M.H.S.); hayhassan@ksu.edu.sa (H.M.A.); 3 Department of Physics and Chemistry, Faculty of Science, Shaqra Univeristy, Shaqra 15572, Saudi Arabia; malmuwayhi@su.edu.sa; 4 Department of Chemistry, College of Science, King Saud University, Riyadh 11451, Saudi Arabia; hulhaque@ksu.edu.sa

**Keywords:** antioxidant system, dehydration stress, *Eruca sativa*, hydrogen sulfide, osmolytes

## Abstract

Hydrogen sulfide (H_2_S) has emerged as an important signaling molecule and plays a significant role during different environmental stresses in plants. The present work was carried out to explore the potential role of H_2_S in reversal of dehydration stress-inhibited *O*-acetylserine (thiol) lyase (OAS-TL), l-cysteine desulfhydrase (LCD), and d-cysteine desulfhydrase (DCD) response in arugula (*Eruca sativa* Mill.) plants. Dehydration-stressed plants exhibited reduced water status and increased levels of hydrogen peroxide (H_2_O_2_) and superoxide (O_2_^•−^) content that increased membrane permeability and lipid peroxidation, and caused a reduction in chlorophyll content. However, H_2_S donor sodium hydrosulfide (NaHS), at the rate of 2 mM, substantially reduced oxidative stress (lower H_2_O_2_ and O_2_^•−^) by upregulating activities of antioxidant enzymes (superoxide dismutase, peroxidase, and catalase) and increasing accumulation of osmolytes viz. proline and glycine betaine (GB). All these, together, resulted in reduced membrane permeability, lipid peroxidation, water loss, and improved hydration level of plants. The beneficial role of H_2_S in the tolerance of plants to dehydration stress was traced with H_2_S-mediated activation of carbonic anhydrase activity and enzyme involved in the biosynthesis of cysteine (Cys), such as OAS-TL. H_2_S-treated plants showed maximum Cys content. The exogenous application of H_2_S also induced the activity of LCD and DCD enzymes that assisted the plants to synthesize more H_2_S from accumulated Cys. Therefore, an adequate concentration of H_2_S was maintained, that improved the efficiency of plants to mitigate dehydration stress-induced alterations. The central role of H_2_S in the reversal of dehydration stress-induced damage was evident with the use of the H_2_S scavenger, hypotaurine.

## 1. Introduction

Climate change is the main factor triggering various environmental stresses, affecting agriculture productivity in almost all the areas of the world. Being sessile in nature, plants are always exposed to these stresses, such as salinity, metal stress, drought stress/dehydration stress, high and low temperature stress, UV radiation, among others. Of these, drought stress, a slow-onset hazard, has been considered as the most devastating, causing severe losses to crop plants in arid and semi-arid regions of the world [1]. Due to global warming, high temperature, low rainfall, high light intensity, dry wind, and fast evaporation of water from soil, cause drought stress [2]. As per agricultural norms, drought can be defined as insufficient soil moisture to meet the needs of a particular crop at a particular time [3]. Prolonged or frequent periods of drought can lead to poor soil fertility that may result in irreversible stage of desertification, unless preventive measures are implemented. Cumulative effects of drought culminate in reduced crop production and loss of livestock that makes agriculture a high-risk endeavor, and can stifle investment, pushing the country into a cycle of underproduction, low income, and persistent poverty. Drought affects the agriculture sector disproportionately, relative to other sectors, and the severity of economic impact of drought is growing. It has been estimated that 83 percent of all the damage and loss to agriculture were caused by drought alone [3]. Between 2005 and 2015, drought caused 30 percent of agricultural loss in developing countries, which amounted to over USD 29 billion, and caused losses of over 11 million human lives, and more than 2 billion have been affected by drought since 1900 [3]. 

The onset of dehydration stress (DS) is the hallmark of drought, that disrupts water relations and suppresses water use efficiency of plants, setting up a condition of water stress. It is well documented that dehydration is a multidimensional stress that causes several changes at morphological, physiological, biochemical, and molecular levels in plants. The mechanisms involved in tolerance of plant to DS vary from plant to plant, and even within species [4,5]. Today, it is very important to develop new tolerant genotypes, and to explore suitable mechanisms which are involved in the tolerance of plant to DS. Under low water conditions, large amounts of reactive oxygen species (ROS) are formed, which dramatically affects entire metabolism of the plant. Although, ROS play a dual role in plants during abiotic stresses, but this dual effect depends on the cellular concentrations [6]. At lower concentrations, ROS act as stress-signaling molecules, and assist the cellular system in transmitting defense responses [6]. On the contrary, excessive accumulation of ROS—up to phytotoxic levels—is exceptionally fatal, due to oxidative stress. The dual role of ROS is governed by their generation and scavenging—a disproportion between these two processes will lead to either excessive accumulation or reduced availability of ROS, that may cause oxidative stress or interrupted signaling system, respectively. Overproduction of ROS causes damage to cellular membranes and macromolecules [7,8,9]. Dehydration stress inhibits plant growth and development by decreasing cell division, elongation, and differentiation due to deprived water status in plant cells, altered enzymes activities, and decreased photosynthesis [2,10,11]. Dehydration stress is one of the main reasons for photosynthesis inhibition by altering stomatal conductance and reducing the supply of CO_2_ to mesophyll tissues and, also, by damaging the key enzyme of Calvin cycle, i.e., ribulose-1,5-bisphosphate carboxylase/oxygenase [2,12,13]. Water deficit in plants also disturbs respiration, translocation, ion uptake, synthesis of protein, amino acids, and carbohydrates, nutrient assimilation and growth regulators [2,4,14]. 

Plants resist DS, caused by soil moisture deficit, by adopting two major strategies: dehydration avoidance and dehydration tolerance [15,16,17,18]. Dehydration avoidance is the capacity of plants to avoid dehydration penetrating the plant tissues and cells by reducing water loss (by a layer of epicuticular wax), or maintaining water uptake through developing a deeper root system, whereas dehydration tolerance occurs when dehydration enters the plant tissues and involves, e.g., turgor maintenance by osmotic adjustment in the plant cells, which allows the plants to function at a lower plant water potential. 

Tolerance is one of the main strategies by which plants can adapt to limited water availability. Plants execute these resistance strategies by modulating a number of physiological and biochemical processes comprised of a network of various kinds of defense systems. For instance, stressed plants accumulate compatible solutes, such as sugars, proline (Pro), and glycine betaine (GB), which maintain osmotic adjustment and assist water uptake in stressed plants. Plants counter oxidative stress through activating antioxidant enzymes, such as catalase (CAT), superoxide dismutase (SOD), and peroxidase (POX). These antioxidant enzymes continuously scavenge ROS and maintain them at normal levels, even under stress conditions. However, excessive accumulation of ROS occurs when the rate of ROS production exceeds the rate of scavenging, and results in oxidative stress. The instantaneous activation of these defense systems, in response to stress stimuli, is carried out by a signaling cascade involving various signaling molecules. However, well-timed and accurate activation of the defense system prior to the commencement of damage is vital for the survival of plants under stressful environmental conditions. Therefore, it is highly desirable to innovate ways that could enhance the inbuilt capacity of plants to counter the detrimental effects of dehydration stress.

In recent years, hydrogen sulfide (H_2_S) has gained substantial attention after its quantification in plants by Wilson et al. [19] and Rennenberg et al. [20]. H_2_S acts as an important signaling molecule during plant response to biotic and abiotic stresses [21,22]. Moreover, H_2_S also acts as an antioxidant, and improves the performance of plants under stress conditions [23,24,25]. H_2_S plays a significant role in various physiological and biochemical processes, including seed germination, morphogenesis, photosynthesis, and flowering [26,27,28]. Plants synthesize H_2_S by degrading cysteine (Cys) through l-cysteine desulfhydrase (LCD; EC 4.4.1.1) and d-cysteine desulfhydrase (DCD; EC 4.4.1.15) enzymes. Plants synthesize Cys from *O*-acetylserine (OAS) and sulfide by the action of *O*-acetylserine (thiol) lyase (OAS-TL; EC 2.5.1.47) enzyme.

*Eruca sativa* Mill. belongs to the Brassicaceae family, is commonly known as rocket or white pepper, green rocket, true rocket, rocket salad, jarjeer, or arugula, and is distributed all over the world. It has high medicinal values and is a rich source of health-promoting agents, such as calcium, magnesium, iron, potassium, sodium, beta-carotene, dietary fiber, vitamins, glucosinolates, and flavonoids [29,30,31,32]. It is widely used as an aphrodisiac in Arab countries. Oil of this plant is used in industries for making lubricant, soap, illuminating agent, and medicines [33]. 

Although several studies have been carried out on the role of H_2_S in plants, scant and dubious information is available on the role of H_2_S in plants, as compared to in the animal system. Therefore, the present investigation was carried out to assess the role of H_2_S in the tolerance of arugula against DS by suppressing oxidative damage. Also, the function of H_2_S was elucidated in the regulation of carbonic anhydrase (CA) activity, Cys pathway, and antioxidant system, which was evident with the use of the H_2_S scavenger, hypotaurine (HT).

## 2. Results

### 2.1. Leaf Relative Water Content (LRWC), Rate of Water Loss, Electrolyte Leakage, and Thiobarbituric Acid Reactive Substances (TBARS)

**Figure 1:**
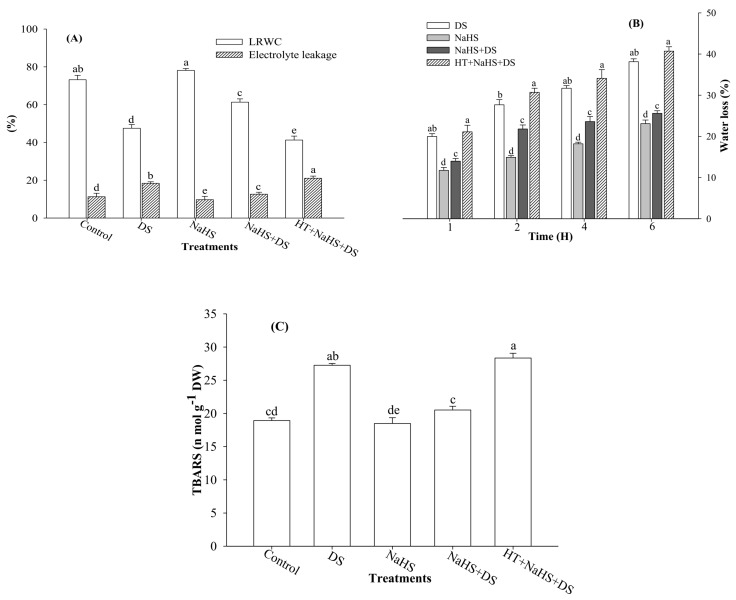
Effect of hydrogen sulfide and dehydration stress on leaf relative water content (LRWC) and electrolyte leakage (**A**), rate of water loss (**B**), and thiobarbituric acid reactive substances (TBARS) (**C**) in arugula. An average of three determinations is presented, with bars indicating SE. Bars followed by the same letter do not differ statistically at *p* < 0.05 (DMRT). (DDW (Control), dehydration stress (DS), 2 mM sodium hydrosulfide (NaHS), 1 mM hypotaurine (HT)).

The results show that plants exposed to DS exhibited a reduction of 35.1% in LRWC over the control (Figure 1A). Moreover, a concomitant increase in the rate of water loss was recorded with the passage of time from 1 to 6 h (Figure 1B). However, addition of the H_2_S donor, NaHS, protected the seedlings from DS and improved LRWC by 29.0%, as compared with the stressed seedlings (Figure 1A). Similar results were also reported for the rate of water loss. For instance, at 4 h, the rate of water loss in dehydration-stressed seedlings was 31.7%, while the rate of water loss was 23.6% in dehydration-stressed seedlings treated with H_2_S (Figure 1B). On the other hand, addition of HT, an H_2_S scavenger, to the incubation medium suppressed the effect of H_2_S on dehydration, and LRWC was further reduced to 13.1% and rate of water loss was increased by 38.5% in stressed seedlings treated with H_2_S (Figure 1A,B). 

Dehydration-stressed seedlings showed an increase of 63.2% in electrolyte leakage as compared with the control (Figure 1A), whereas application of H_2_S to stressed seedlings reduced electrolyte leakage by 31.4% as compared with dehydration-stressed plants. On the contrary, an addition of HT countered the impact of H_2_S and, again, caused a significant increase in electrolyte leakage to a level higher than the dehydration-suffered seedlings (Figure 1A).

Dehydration-stressed seedlings exhibited 44.0% higher value for TBARS as compared with the control (Figure 1C). However, H_2_S reduced the level of TBARS by 24.8% in stressed plants in comparison with dehydration-stressed seedlings not treated with H_2_S (Figure 1C). On the contrary, the H_2_S scavenger, HT, mutated the stress, alleviating the impact of H_2_S and further caused a significant increase in TBARS, reaching the value statistically close to the value recorded from dehydration-stressed seedlings (Figure 1C).

### 2.2. Hydrogen Peroxide (H_2_O_2_) and Superoxide (O_2_^•−^) Content

**Figure 2:**
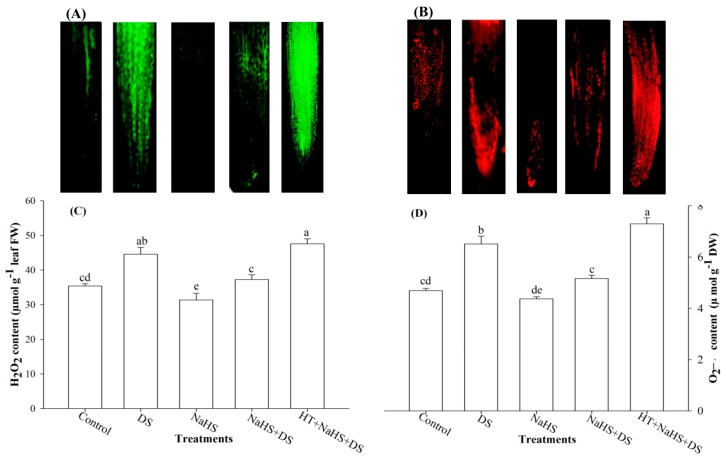
Fluorescence microscopy imaging of reactive oxygen species (ROS): H_2_O_2_ production (H_2_O_2_-dependent DCF-DA fluorescence) in root (**A**), O_2_^•−^ production (O_2_^•−^ dependent DHE fluorescence) in root (**B**). Effect of hydrogen sulfide and dehydration stress on H_2_O_2_ content (**C**) and O_2_^•−^ content (**D**) in arugula. Average of three determinations is presented, with bars indicating SE. Bars followed by the same letter do not differ statistically at *p* < 0.05 (DMRT). (DDW (Control), dehydration stress (DS), 2 mM sodium hydrosulfide (NaHS), 1 mM hypotaurine (HT)).

The visual effect of dehydration stress and H_2_S on the production of ROS in roots was tested by assessing in situ formation of H_2_O_2_ and O_2_^•−^ in roots using a DCF-DA and DHE fluorescence probe, respectively. Roots of the seedlings exposed to DS exhibited sharper green (with DCF-DA probe) and red fluorescence (with DHE probe) signal than the control plants. However, a lower fluorescence signal was observed in the dehydration-suffered roots treated with H_2_S donor, NaHS, as compared with the stressed plants not treated with NaHS, whereas fluorescence signals again sharpened in the roots of stressed plants that received H_2_S scavenger HT along with NaHS (Figure 2A,B)

Dehydration stress-induced generation of ROS was measured in terms of H_2_O_2_ and O_2_^•−^ content. Perusal of the data shows that dehydration-stressed plants generated 25.9% and 38.8% more H_2_O_2_ and O_2_^•−^ content, respectively, as compared with the control (Figure 2C,D). However, H_2_S showed an inhibitory effect on the generation of these ROS. Application of H_2_S to stressed seedlings caused a reduction of 16.4% and 20.7% in H_2_O_2_ and O_2_^•−^ content, respectively, compared with stressed samples. By contrast, the addition of HT to incubation medium suppressed the effect of H_2_S on dehydration-stressed plants, that further elevated the levels of H_2_O_2_ and O_2_^•−^ content (Figure 2C,D).

### 2.3. Proline (Pro) and Glycine Betaine (GB) Content

**Figure 3:**
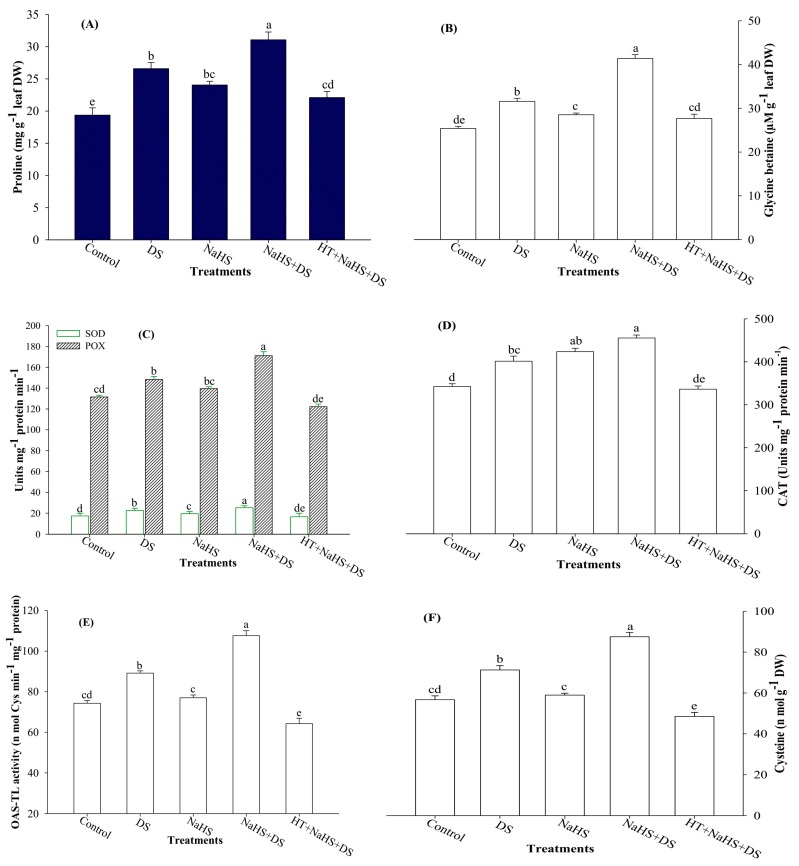
Effect of hydrogen sulfide and dehydration stress on proline (Pro) content (**A**), glycine betaine (GB) content (**B**), activities of SOD and POX (**C**), activity of CAT (**D**), activity of OAS-TL (**E**), and cysteine content (**F**) in arugula. Average of three determinations is presented, with bars indicating SE. Bars followed by the same letter do not differ statistically at *p* < 0.05 (DMRT). (DDW (Control), dehydration stress (DS), 2 mM sodium hydrosulfide (NaHS), 1 mM hypotaurine (HT)).

In the present study, dehydration-stressed plants accumulated 37.3% and 24.4% more Pro and GB content, respectively, than the control plants (Figure 3A,B). Moreover, application of H_2_S further elevated Pro and GB content by 16.9% and 31.1%, respectively, as compared with dehydration-stressed plants, whereas the presence of HT in the incubation medium (HT + NaHS + DS) reversed the effect of H_2_S on these osmolytes and, as a result, Pro and GB content dropped to 16.9% and 12.4%, respectively, as compared with the stressed plants (Figure 3A,B).

### 2.4. Activities of Antioxidant Enzymes

Higher activities of antioxidant enzymes (SOD, POX, and CAT) were recorded in stressed seedlings as compared with the control plants (Figure 3C,D). Furthermore, an additional increase of 17.5%, 15.4%, and 13.5% was recorded in SOD, POX, and CAT, respectively, in dehydration-stressed seedlings supplemented with H_2_S donor NaHS. On the other hand, the inclusion of HT to NaHS-treated stressed plants (HT + NaHS + DS) suppressed the activities of SOD, POX, and CAT by 23.0%, 17.6%, and 16.2%, respectively, compared with dehydration-stressed plants (Figure 3C,D).

### 2.5. Activity of O-Acetylserine (Thiol) Lyase (OAS-TL) Enzyme and Cysteine (Cys) Content

Perusal of the data shows that DS enhanced the activity of Cys-synthesizing enzyme OAS-TL, as well as Cys content by 20.0% and 25.6%, respectively, when compared with the control (Figure 3E,F). In addition, treatment of dehydration-stressed plants with NaHS (NaHS + DS) further enhanced these parameters to the highest level. The treatment of NaHS + DS enhanced OAS-TL activity and Cys content by 20.7% and 22.9%, respectively, compared with the stressed plants. On the contrary, the inclusion of H_2_S scavenger HT (HT + NaHS + DS) slowed down the activity of OAS-TL by 27.9%, which was 20.7% higher when HT was not present (NaHS + DS) in the incubation medium (Figure 2E,F). A similar inhibitory effect of HT was also noticed on Cys content, which exhibited a 31.9% lower value than the stressed seedlings (Figure 3F). 

### 2.6. Activities of LCD and DCD Enzymes and H_2_S Content

**Figure 4:**
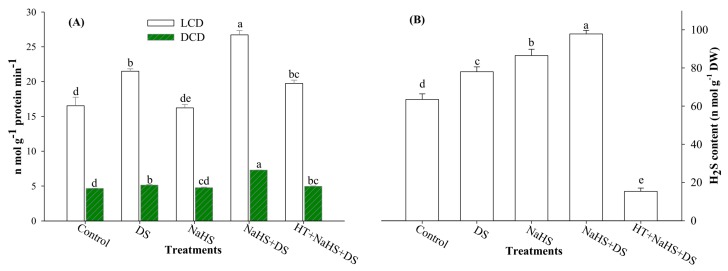
Effect of hydrogen sulfide and dehydration stress on the activities of LCD and DCD enzymes (**A**), and H_2_S content (**B**) in arugula. Average of three determinations is presented, with bars indicating SE. Bars followed by the same letter do not differ statistically at *p* < 0.05 (DMRT). (DDW (Control), dehydration stress (DS), 2 mM sodium hydrosulfide (NaHS), 1 mM hypotaurine (HT)).

It is evident from Figure 4A,B that DS induced the activities of LCD and DCD enzymes, and H_2_S content. Dehydration-stressed samples showed 30.0% and 10.3% higher activities of LCD and DCD enzymes, respectively, compared with the control. Likewise, an increase of 19.7% was also recorded in H_2_S content of dehydration-stressed plants. Additionally, exogenous application of H_2_S to dehydration-stressed seedlings (NaHS + DS) further elevated the activities of LCD and DCD enzymes and H_2_S content by 24.3%, 41.9%, and 28.7%, respectively, compared with the stressed plants (Figure 3A,B). Nevertheless, the addition of HT to H_2_S-supplemented dehydration-stressed plants (HT + NaHS + DS) suppressed the LCD and DCD activities, and H_2_S content. HT scavenged 79.8% H_2_S in H_2_S-supplemented stressed seedlings (NaHS + DS) as compared with dehydration-stressed seedlings (Figure 4B). It is worth mentioning here that impact of HT on H_2_S was more prominent than on the activities of LCD and DCD enzymes.

### 2.7. Photosynthetic Pigments and Carbonic Anhydrase (CA) Activity

As evident from previous studies, DS causes destruction of photosynthetic pigments. A similar trend was also recorded in the present investigation. Dehydration-suffered plants showed a 25.5%, 46.9%, and 32.9% decrease in Chl *a*, Chl *b*, and total Chl content, respectively, as compared with the control (Table 1). However, stressed plants showed an increase of 40.1% in Chl *a*/*b* ratio, as compared with control. Incubation of dehydration-stressed plants with H_2_S improved Chl *a*, Chl *b*, and total Chl content by 29.2%, 66.7%, and 39.4%, respectively, but H_2_S decreased Chl *a*/*b* ratio by 22.7% compared with the stressed seedlings. Plants exposed to DS exhibited a 12.8% increase in carotenoid content, as compared with the control. Furthermore, dehydration-stressed plants treated with H_2_S exhibited a further increase of 15.2% in total carotenoid content as compared with the stressed plants that did not receive H_2_S treatment (Table 1). Conversely, the presence of HT, along with exogenous H_2_S in incubation medium, created stress-like conditions, as reflected by the reduced values of all these parameters, except Chl *a*/*b* ratio, which showed an increase when compared with dehydration-stressed plants without H_2_S (Table 1).

Results showed that DS upregulated carbonic anhydrase (CA) activity and caused an increase of 16.3% compared with the control plants (Table 1). In addition, stressed plants, when treated with NaHS (NaHS + DS), exhibited a further increase of 15.5% in CA activity than the dehydration-stressed plants that did not receive H_2_S. On the other hand, addition of HT (HT + NaHS + DS) invalidated the effect of H_2_S and caused a reduction in the activity of CA (Table 1).

## 3. Discussion

It is well established that DS has multiple effects on plants. Reduction in water status is the hallmark of DS. Therefore, keeping an eye on the water status of plants would be amongst one of the strategies to save crop plants from DS. To test the effect of dehydration on water status, we measured LRWC (Figure 1A). Plants under DS showed lower values for LRWC and an enhanced rate of water loss over the time. Consistent DS leads to the creation of hyperosmotic stress, which affects water relations and causes loss of turgor [34] that reduces water uptake capacity of plants, as reflected by lower LRWC (Figure 1A). To evaluate the water-retaining capacity of plants, we performed a water loss assay. Dehydration-stressed seedlings showed a parallel increase in the rate of water loss from 1 to 6 h of estimation (Figure 1B). Plants counter osmotic stress through osmotic adjustment, which is normally accomplished by the accumulation of osmolytes, such as Pro and GB, that maintain normal water status in plants under abiotic stress [35,36]. Enhanced levels of Pro and GB were recorded in the same dehydration-stressed plants in which lower LRWC and a higher rate of water loss was noticed. This indicated that enhanced levels of Pro and GB were not sufficient to maintain the normal hydration level of the plants. However, the presence of H_2_S in the growth medium further enhanced Pro and GB content (Figure 3A,B) to a level that vigorously countered dehydration-induced osmotic stress, as reflected by enhanced LRWC and a lower rate of water loss in stressed plants (Figure 1A,B). An increment in Pro and GB content by H_2_S can be explained on the basis of the role of H_2_S in enhancing the activities of key enzymes responsible for Pro and GB synthesis [37,38]. Furthermore, H_2_S has been shown to increase the expression levels of abscisic acid (ABA) biosynthesis and reactivation genes [39], leading to stomatal closure [40]. However, H_2_S inhibits ethylene synthesis through suppressing the activity of 1-aminocyclopropane-1-carboxylic acid oxidase [41]. An increase in ABA and decrease in ethylene has been reported to be associated with a decrease in transpiration rate and leaf water potential [42], which improved the water-retaining capacity of plants, as witnessed by improved LRWC and reduced rate of water loss (Figure 1A,B). Alleviation of DS and an increase in relative water content due to H_2_S treatment was also reported by García-Mata and Lamattina [43], and Chen et al. [44]. On the contrary, H_2_S scavenger HT mutated the effect of H_2_S that resulted in suppressed accumulation of Pro and GB content (Figure 3A,B) and, in turn, lower LRWC (Figure 1A), and a higher rate of water loss (Figure 1B) was recorded.

Continued drought results in severe adverse effects as it creates oxidative stress through overproduction of ROS. Excessive generation of ROS causes peroxidation of membrane lipids and damage to cellular compartments, proteins, and RNA and DNA molecules [6,45]. The present study also shows higher levels of ROS (H_2_O_2_ and O_2_^•−^ content) (Figure 2A–D) coupled with increased electrolyte leakage and TBARS in stressed seedlings (Figure 1A,C). Plants counter oxidative stress by activating their antioxidant system. Antioxidant enzyme SOD converts O_2_^•−^ radicals to H_2_O_2_, while POX and CAT convert H_2_O_2_ into water and oxygen. Results show that DS enhanced the activities of antioxidant enzymes (SOD, POX, and CAT), but increased levels of antioxidant enzymes proved weak in detoxifying ROS, as shown by increased values of H_2_O_2_ and O_2_^•−^ content (Figure 2A–D). This indicates that under DS, the antioxidant system was imbalanced, and the rate of ROS generation surpassed the rate of their scavenging. However, H_2_S donor NaHS further enhanced the activities of antioxidant enzymes to a level adequate to counter excessive generation of ROS that ultimately resulted in improved LRWC, and a reduction in water loss, electrolyte leakage, and TBARS [27,28,39,46] (Figure 1A–C). A role for H_2_S in reducing oxidative stress was further confirmed when we used the H_2_S scavenger, HT. Addition of HT to dehydration-stressed plants, supplemented with H_2_S, reverted the effect of H_2_S that, again, increased the generation of ROS and resulted in a condition similar to DS, as shown by enhanced electrolyte leakage and TBARS (Figure 1A,C), and also shown in the visual detection of ROS (Figure 2A,B). Our results are in agreement with the findings of Luo et al. [38], Shi et al. [47], and Fu et al. [48], who observed that exogenous application of NaHS decreased H_2_O_2_ and O_2_^•−^ content and electrolyte leakage, and induced stress tolerance, while HT exhibited a reverse effect. 

**Figure 5:**
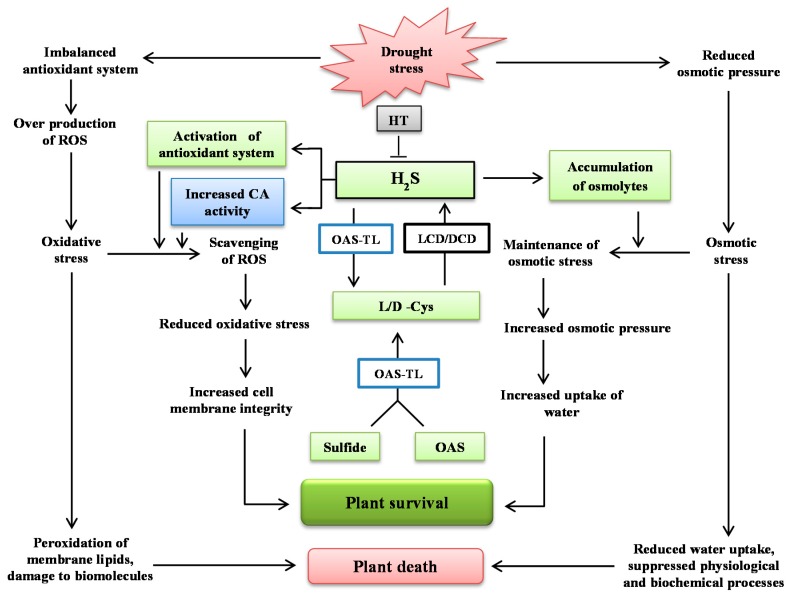
Depiction of the role of H_2_S in the protection of plants against dehydration stress. CA: carbonic anhydrase; DCD: d-cysteine desulfhydrase; HT: hypotaurine; l/d-Cys: l/d-cysteine; LCD: l-cysteine desulfhydrase; OAS: *O*-acetylserine; OAS-TL: *O*-acetylserine (thiol) lyase.

It is well known that Cys is synthesized by incorporation of reduced sulfur to *O*-acetylserine in a reaction catalyzed by the enzyme OAS-TL (Figure 5). Cys serves as a sulfur donor for synthesis of S-containing compounds [49], and as a precursor of various antioxidants and defense compounds [50], and induces tolerance to various stresses [51]. Results show that DS enhanced the activity of OAS-TL, that resulted in enhancement of Cys content (Figure 3E,F). As discussed in the preceding paragraph, DS also enhanced H_2_O_2_ and O_2_^•−^ content, electrolyte leakage, and TBARS. It shows that seedlings tried to counter DS by increasing Cys synthesis, but an increase in Cys was not enough to counter available ROS. Nevertheless, application of NaHS to stressed seedlings further enhanced OAS-TL activity and Cys content coupled with a decrease in water loss, ROS, electrolyte leakage, and TBARS, and an increase in LRWC (Figure 1A–C and Figure 2A–D). On the other hand, treatment with HT reversed the effect of NaHS, that resulted in enhanced levels of water loss, ROS, electrolyte leakage, and TBARS, and reduced LRWC. Thus, it is clear from the results that exogenous application of NaHS enhances Cys-synthesizing enzyme and Cys content, that directly or indirectly gives protection against stress-induced constraints.

It is evident from the results shown in Figure 4A,B that DS significantly enhanced the activities of H_2_S-synthesizing enzymes LCD and DCD, and H_2_S content. The enzymes LCD and DCD synthesize H_2_S from l-Cys and d-Cys, respectively (Figure 5) [52,53]. Therefore, an endogenous level of H_2_S directly depends on the availability of Cys. In the present study, DS enhanced OAS-TL, LCD, and DCD activities, and Cys and H_2_S content, but seedlings failed to counter imposed DS. Moreover, treatment with H_2_S further enhanced these parameters. Enhanced levels of H_2_S proved effective in protecting plants against DS via enhancing the accumulation of osmolytes and activities of antioxidant enzymes, as witnessed by improved hydration level and reduced water loss, electrolyte leakage, and TBARS [25,54]. On the other hand, the addition of HT to the incubation medium scavenged H_2_S to a considerable limit, and suppressed the activities of LCD and DCD enzymes (Figure 4A,B). 

Photosynthetic pigments, the key light harvesting devices of plants, are responsible for the conversion of light energy to usable chemical energy for all the creatures of planet Earth. Plants under DS exhibited reduced levels of the studied photosynthetic pigments viz. Chl *a*,*b*, and total Chl content with the exception of Chl *a*/*b* ratio and carotenoid content which showed an increase compared with control plants (Table 1). A decrease in Chl content by DS has already been reported by several scientists [25,34,55,56]. A decline in Chl content was probably the result of an inhibitory effect of DS on Chl biosynthetic intermediates and decrease in Chl biosynthesis enzymes [57]. Moreover, DS-induced overproduction of ROS might have caused photo-oxidative damage to Chl, as shown by reduced Chl *a,b* and total Chl content (Table 1). It is interesting to note, here, that dehydration-stressed plants show increased Chl *a*/*b* ratio than the non-stressed control plants. This indicates that Chl *b* is more susceptible to dehydration-induced damage than Chl *a*, as shown by higher decrease in Chl *b* content (46.9%) than Chl *a* (25.5%) [58]. However, treatment of plants with H_2_S donor NaHS overcame the effect of DS and reduced the level of ROS through enhancing the activity of antioxidant enzymes (Figure 3C,D). Reduction in ROS gave protection to cellular membranes that resulted in the suppression of electrolyte leakage and TBARS (Figure 1A,C). As a result, H_2_S created a condition that could be suitable for the biosynthesis of Chl and resulted in improved Chl content (Table 1) [59,60,61]. Besides antioxidant enzymes in plants, carotenoids act as first line of defense in chloroplast against O_2_ toxicity, especially singlet oxygen (^1^O_2_). Dehydration stress induced carotenoids, and a further increase was also noticed in H_2_S-treated stressed plants that might have prohibited oxidative stress in the chloroplast, leading to the normal synthesis of Chl even under stressful conditions. On the contrary, the addition of H_2_S scavenger, HT, validated the role of H_2_S by reducing Chl and carotenoid content (Table 1). 

Carbonic anhydrase (CA) is a chloroplast-localized enzyme that catalyzes the reversible hydration of CO_2_, and maintains its continuous supply to Rubisco, a key enzyme responsible for the fixation of CO_2_. In addition to its direct role in photosynthesis, expression of CA is related with environmental stresses, and CA activity is upregulated under stresses [62]. An increase in CA activity was recorded in dehydration-stressed plants; moreover, treatment with NaHS further enhanced CA activity in non-stressed, as well as stressed, plants (Table 1). Increased activity of CA controls ROS levels during DS, and helps the cells to become more resistant to cytotoxic concentrations of H_2_O_2_ [63,64]. Therefore, it can be explained that H_2_S-induced reduction in ROS was due to enhanced levels of SOD and carotenoids, that converted oxygen radicals to H_2_O_2_ and, further, the level of H_2_O_2_ was regulated by the joint action of CA, POX, and CAT. The effect of reduced concentrations of ROS is also witnessed by enhanced LRWC and reduced water loss, electrolyte leakage, and TBARS in treated plants (Figure 1A–E). This is the first report regarding the response recorded from the effect of H_2_S in the reduction of ROS generation by the enhanced activity of CA under DS. 

## 4. Materials and Methods

### 4.1. Plant Culture and Treatments

Healthy and uniform seeds of arugula (*Eruca sativa* Mill.) were surface-sterilized with sodium hypochlorite (1%), followed by repeated washing with double distilled water (DDW). The seeds were sown 2 cm deep in plastic pots (20 cm diameter and 20 cm height) containing a mixture of soil/vermiculite (1:1). The pots were kept under natural illuminated conditions with an average day/night temperature of 28/10 ± 3 °C, and were well-watered (100% field capacity). After one week, the seedlings were exposed to 2 mM sodium hydrosulfide (NaHS), 1 mM hypotaurine (HT), and dehydration stress (DS). Dehydration stress was imposed by maintaining the water level in soil at 30% through withholding the water supply. During the dehydration period, soil relative water content was monitored daily, and DS was continued up to 10 days when the desired level of soil relative water content (30%) was attained. Plants (control) which were not exposed to dehydration were irrigated with DDW throughout the period of dehydration. The treatments were given as (i) DDW: Control, (ii) Dehydration stress (DS), (iii) 2 mM NaHS + DDW: (NaHS), (iv) 2 mM NaHS + DS: (NaHS + DS) and (v) 1 mM HT + 2 mM NaHS + DS: (HT + NaHS + DS). NaHS was used as H_2_S donor, while HT was used as H_2_S scavenger. The design of the experiment was simple-randomized, with three replicates per treatment. After 10 days of treatments, the seedlings were uprooted carefully, and the response of seedlings to the treatments was further evaluated. 

### 4.2. Estimation of Leaf Relative Water Content (LRWC), Electrolyte Leakage, and Rate of Water Loss

After 10 days of dehydration treatment, the fresh weight (FW) of the leaves was measured. To determine turgid weight (TW), the leaves were immersed in DDW inside the covered Petri dish. After 4 h, the water from leaf surface was wiped with blotting paper, and TW was recorded. Finally, the leaf samples were oven-dried at 80 °C for 24 h, and then the dry weight (DW) was determined. Leaf relative water content was estimated according to Yamasaki and Dillenburg [65], using the following formula:

LRWC (%) = (FW−DW)/(TW−DW) × 100

The effect of DS and H_2_S on membrane permeability was assessed by measuring electrolyte leakage by the method of Lutts et al. [66]. After 10 days of treatment, electrical conductivity of the leaves incubated with DDW for 24 h (EC1), and leaves autoclaved at 120 °C for 20 min (EC2), were measured. The electrolyte leakage (%) was calculated as ((EC1/EC2) × 100).

To determine the rate of water loss, treated leaves were placed on a filter paper at room temperate under white fluorescent light. These leaves were weighed periodically at an interval of 1 h for up to 6 h. The rate of water loss was expressed as a percentage of the control.

### 4.3. Detection of Hydrogen Peroxide (H_2_O_2_) and Superoxide (O_2_^•−^) in Roots

In the roots of experimental seedlings, H_2_O_2_ and O_2_^•−^ were visualized according to the method of Rodriguez-Serrano et al. [67] using fluorescence probes 2′,7′-dichlorofluorescein diacetate (DCF-DA) for H_2_O_2_ and dihydroethidium (DHE) for O_2_^•−^. A fluorescence microscope (Eclipse Ni-U, Nikon, Tokyo, Japan) was used to capture signals of DCF-DA at the excitation wavelength of 480 nm and emission wavelength of 530 nm, whereas DHE signals were captured at the excitation and emission wavelengths of 490 and 520 nm, respectively.

### 4.4. Estimation of H_2_O_2_ and O_2_^•−^ Content 

Hydrogen peroxide (H_2_O_2_) in the leaves was determined using 10 M potassium phosphate buffer and 1 M potassium iodide [68]. The absorbance of the samples was read at 390 nm. The H_2_O_2_ content was measured by comparing with a standard curve, and was expressed as μmol g^−1^ leaf FW.

Superoxide (O_2_^•−^) content was determined by adopting the method of Elstner and Heupel [69] with some modifications. The absorbance was measured at 530 nm. The content of O_2_^•−^ was calculated by comparing with a standard curve.

### 4.5. Analysis of Lipid Peroxidation

Peroxidation of lipids was tested by measuring the thiobarbituric acid reactive substances (TBARS), as described by Cakmak and Horst [70]. The absorbance of the supernatant was measured at 532 nm and 600 nm. The values were corrected for non-specific turbidity by subtracting the absorbance. TBARS content was expressed as nmol g^−1^ DW.

### 4.6. Determination of Proline (Pro) and Glycine Betaine (GB) Content

Proline (Pro) content was determined based on the reaction of proline with acid ninhydrin [71]. The mixture was extracted with toluene, and the free toluene was quantified at 528 nm using l-proline as standard.

Glycine betaine (GB) content was measured by the method of Grieve and Grattan [72]. Using aqueous extracts of dry-ground material after reaction with KI_2_-I_2_, GB concentration was estimated at 365 nm.

### 4.7. Assay of Antioxidant Enzymes

A crude enzyme extract was prepared prior to determination of activities of antioxidant enzyme. Seedlings were homogenized with three volumes (*w*/*v*) of an ice-cold extraction buffer (1 mM MgCl_2_, 1.5% (*w*/*w*) polyvinylpyrrolidone, 1 mM EDTA, and 50 mM Tris-HCl, pH 7.8). The supernatant was collected after centrifugation of homogenate at 15,000×*g* at 4 °C for 20 min. The supernatant was stored at −20 °C, and was used as the crude extract for the assay of activities of superoxide dismutase (SOD), peroxidase (POX), and catalase (CAT).

Superoxide dismutase (SOD; EC 1.15.1.1) activity was determined by estimating the enzymes’s capacity of inhibiting the photochemical reduction of nitroblue tetrazolium [73]. The absorbance of the solution was measured at 560 nm. 

Peroxidase (POX; EC 1.11.1.7) was assayed using the method of Upadhyaya et al. [74]. The reaction mixture contained 10–20 μL of enzyme extract, 1 mL of 1% hydrogen peroxide, 2.5 mL of 50 mM potassium phosphate buffer (pH 6.1), and 1 mL of 1% guaiacol. The escalation in absorbance was read at 420 nm.

Catalase (CAT; EC 1.11.1.6) activity was measured according to Cakmak and Marschner [75]. The reaction mixture was comprised of 0.1 mL enzyme extract, 10 mM H_2_O_2_, and 25 mM sodium phosphate buffer (pH 7.0). A decrease in absorbance due to the decline of extinction of H_2_O_2_ was recorded at 240 nm.

### 4.8. Determination of OAS-TL Enzyme Activity and Cys Content

The activity of *O*-acetylserine (thiol) lyase (OAS-TL; EC 2.5.1.47) and cysteine content was determined according to Gaitonde [76], as explained by Riemenschneider et al. [77] with a few modifications. OASTL activity and Cys content were determined in soluble protein extract. The assay mixture (1 mL) for OAS-TL activity contained 50 mL enzyme extract, 100 mM Tris-HCl (pH 7.5), 5 mM Na_2_S, 33.4 mM dithiotreitol, and 5 mM *O*-acetyl serine (OAS) [78]. The Cys concentration (n mol Cys min^−1^ mg^−1^ protein) was determined by adding Na_2_S to the reaction mixture, and incubated for 30 min at 37 °C, followed by the addition of 1 mL acid ninhydrin reagent [76]. The Cys content was estimated using pure Cys as standard. The absorbance was measured at 560 nm, and the result was expressed as n mol g^−1^ DW.

### 4.9. Measurement of LCD and DCD Enzyme Activities and H_2_S Content

The activities of l-cysteine desulfhydrase (LCD; EC 4.4.1.1) and D-cysteine desulfhydrase (DCD; EC 4.4.1.15) were determined as described by Bloem et al. [79] and Riemenschneider et al. [80], respectively. LCD activity in enzyme extract (extracted in Tris-HCl buffer, pH 9.0) was measured by the release of H_2_S from l-Cys. The absorbance was read at 670 nm. DCD activity was determined by the same method, except that d-Cys was used instead of l-Cys, and the pH of the Tris-HCl buffer was 8.0 instead of 9.0. The standard curve of known concentrations of Na_2_S were prepared for the quantification of H_2_S. 

Concentration of H_2_S in fresh tissues was determined according to Nashef et al. [81]. The assay mixture contained 20 mL of 20 mM 5,5’-dithiobis(2-nitrobenzoic acid) and 1880 μL extraction buffer. The assay mixture was incubated at room temperature for 2 min, and the absorbance was read at 412 nm. The values were compared with the standard curve of Na_2_S solutions of different concentrations.

### 4.10. Estimation of Photosynthetic Pigments and Carbonic Anhydrase (CA) Activity 

The method of Lichtenthaler and Buschmann [82] was used for the estimation of chlorophyll (Chl) and total carotenoid content. The pigment solution of fresh tissue was prepared by grinding the samples with 100% acetone. The optical density of the pigment solution was recorded at 662, 645, and 470 nm to determine Chl *a*, Chl *b*, and total carotenoid content, respectively, using a spectrophotometer.

Carbonic anhydrase (CA; EC 4.2.1.1) activity was estimated according to Dwivedi and Randhawa [83] using phosphate buffer (pH 6.8), and bromothymol blue and methyl red indicators. The reaction mixture was titrated against 0.05 N HCl. The values were expressed as μmol CO_2_ kg^−1^ leaf FW s^−1^.

### 4.11. Statistical Analysis

Data were presented as the means ± SE of three independent replicates. The data were analyzed statistically with SPSS-17 statistical software (SPSS Inc., Chicago, IL, USA). Means were statistically compared by Duncan’s multiple range test (DMRT) at *p* < 0.05% level.

## 5. Conclusions

Dehydration stress enhanced the accumulation of osmolytes and activities of antioxidant enzymes. Concurrently, DS also caused excessive generation of ROS and decrease in hydration level of plants. Therefore, dehydration-induced enhancement of osmolytes and antioxidant enzymes was not capable of providing enough protection against the detrimental effects of DS. However, application of H_2_S donor NaHS induced Cys content via enhancing the activity of OAS-TL enzyme. Exogenous application of H_2_S also induced the activities of LCD and DCD enzymes that synthesized more H_2_S from accumulated Cys, and enhanced the concentration of endogenous H_2_S. An enhanced level of H_2_S accelerated the synthesis of osmolytes and activities of antioxidant enzymes and CA, that enabled the plants to counter the damaging effects of DS. A constructive effect of H_2_S on dehydration-induced impairment was also witnessed by reduced H_2_O_2_ and O_2_^•−^ content, electrolyte leakage, and TBARS, resulting in an improved hydration level, enhanced concentration of photosynthetic pigments, and upregulated CA activity, a key enzyme of photosynthesis (Figure 5). Consequently, it can be concluded that H_2_S alleviated DS by enhancing Cys synthesis and endogenous H_2_S accumulation, that induced osmolyte accumulation and an antioxidant system which enabled the plants to perform normally even under adverse conditions of dehydration (Figure 5). 

## Figures and Tables

**Table 1:** Effect of hydrogen sulfide and dehydration stress on chlorophyll content, total carotenoids, and carbonic anhydrase activity in arugula.

Treatments	Parameters					
	Chl *a* (mg g^−1^ FW)	Chl *b* (mg g^−1^ FW)	Total Chl (mg g^−1^ FW)	Chl *a*/*b*	Total carotenoids (mg g^−1^ FW)	CA activity (μM CO_2_ kg^−1^ leaf FW s^−1^)
Control	1.84 ± 0.04 ^a^	0.96 ± 0.10 ^b^	2.80 ± 0.09 ^ab^	1.92 ± 0.29 ^cd^	3.68 ± 0.19 ^de^	276.40 ± 11.39 ^d^
DS	1.37 ± 0.06 ^d^	0.51 ± 0.04 ^d^	1.88 ± 0.07 ^d^	2.69 ± 0.11 ^b^	4.15 ± 0.06 ^b^	321.58 ± 3.96 ^bc^
NaHS	1.82 ± 0.06 ^ab^	1.07 ± 0.07 ^a^	2.89 ± 0.06 ^a^	1.70 ± 0.09 ^e^	3.82 ± 0.08 ^bc^	336.73 ± 4.96 ^b^
NaHS + DS	1.77 ± 0.03 ^ac^	0.85 ± 0.10 ^c^	2.62 ± 0.08 ^ac^	2.08 ± 0.24 ^c^	4.78 ± 0.11 ^a^	371.27 ± 7.14 ^a^
HT + NaHS + DS	1.25 ± 0.03 ^de^	0.25 ± 0.09 ^e^	1.50 ± 0.10 ^e^	5.00 ± 0.15 ^a^	3.76 ± 0.08 ^bd^	266.49 ± 15.22 ^de^

Values are average ± SE of three independent replicates. Values followed by the same letter within the column do not differ statistically at *p* < 0.05 (DMRT). (DDW (Control), dehydration stress (DS), 2 mM NaHS (NaHS), 1 mM hypotaurine (HT), fresh weight (FW)).
